# Emotional Expression in Children With ASD: A Pre-Study on a Two-Group Pre-Post-Test Design Comparing Robot-Based and Computer-Based Training

**DOI:** 10.3389/fpsyg.2021.678052

**Published:** 2021-07-21

**Authors:** Flavia Lecciso, Annalisa Levante, Rosa Angela Fabio, Tindara Caprì, Marco Leo, Pierluigi Carcagnì, Cosimo Distante, Pier Luigi Mazzeo, Paolo Spagnolo, Serena Petrocchi

**Affiliations:** ^1^Department of History, Society and Human Studies, University of Salento, Lecce, Italy; ^2^Laboratory of Applied Psychology and Intervention, University of Salento, Lecce, Italy; ^3^Department of Clinical and Experimental Medicine, University of Messina, Messina, Italy; ^4^Institute of Applied Sciences and Intelligent Systems, National Research Council, Lecce, Italy; ^5^Faculty of Biomedical Sciences, Università della Svizzera Italiana, Lugano, Switzerland

**Keywords:** new technology, autism spectrum disorder, social skills, emotion recognition, emotion expression, robot, computer, training

## Abstract

Several studies have found a delay in the development of facial emotion recognition and expression in children with an autism spectrum condition (ASC). Several interventions have been designed to help children to fill this gap. Most of them adopt technological devices (i.e., robots, computers, and avatars) as social mediators and reported evidence of improvement. Few interventions have aimed at promoting emotion recognition and expression abilities and, among these, most have focused on emotion recognition. Moreover, a crucial point is the generalization of the ability acquired during treatment to naturalistic interactions. This study aimed to evaluate the effectiveness of two technological-based interventions focused on the expression of basic emotions comparing a robot-based type of training with a “hybrid” computer-based one. Furthermore, we explored the engagement of the hybrid technological device introduced in the study as an intermediate step to facilitate the generalization of the acquired competencies in naturalistic settings. A two-group pre-post-test design was applied to a sample of 12 children (M = 9.33; ds = 2.19) with autism. The children were included in one of the two groups: group 1 received a robot-based type of training (*n* = 6); and group 2 received a computer-based type of training (*n* = 6). Pre- and post-intervention evaluations (i.e., time) of facial expression and production of four basic emotions (happiness, sadness, fear, and anger) were performed. Non-parametric ANOVAs found significant time effects between pre- and post-interventions on the ability to recognize sadness [*t*_(1)_ = 7.35, *p* = 0.006; pre: M (ds) = 4.58 (0.51); post: M (ds) = 5], and to express happiness [*t*_(1)_ = 5.72, *p* = 0.016; pre: M (ds) = 3.25 (1.81); post: M (ds) = 4.25 (1.76)], and sadness [*t*_(1)_ = 10.89, *p* < 0; pre: M (ds) = 1.5 (1.32); post: M (ds) = 3.42 (1.78)]. The group^*^time interactions were significant for fear [*t*_(1)_ = 1.019, *p* = 0.03] and anger expression [*t*_(1)_ = 1.039, *p* = 0.03]. However, Mann–Whitney comparisons did not show significant differences between robot-based and computer-based training. Finally, no difference was found in the levels of engagement comparing the two groups in terms of the number of voice prompts given during interventions. Albeit the results are preliminary and should be interpreted with caution, this study suggests that two types of technology-based training, one mediated *via* a humanoid robot and the other *via* a pre-settled video of a peer, perform similarly in promoting facial recognition and expression of basic emotions in children with an ASC. The findings represent the first step to generalize the abilities acquired in a laboratory-trained situation to naturalistic interactions.

## Introduction

Emotions are social and dynamic processes, and they serve as early mediators of communication during childhood (Ekman, 1984; Eisenberg et al., 2000; Davidson et al., 2009). Emotions are mental states that, at the same time, define social interactions and are determined by them (Halberstadt et al., 2001). When children express emotions, they convey a message or a need to others who recognize and understand them in order to respond appropriately to the children. Similarly, the understanding of the emotions of others allows children to develop social skills and learn how to become a socially competent partner (Marchetti et al., 2014). Furthermore, emotional competence is one of the pivotal components of many social processes, appropriate inter-individual interactions, and adaptive behaviors (Schutte et al., 2001; Lopes et al., 2004, 2005; Buckley and Saarni, 2006; Nuske et al., 2013). A demonstration of the crucial role of emotional competence as a social skill derives by examining individuals with well-known impairments in social functioning. One such group is composed of individuals with an autism spectrum condition [henceforth ASC (American Psychiatric Association, 2013)], a neurodevelopmental disorder characterized by two core symptoms: social communication deficits (diagnostic criterion A) and a pattern of repetitive and restricted behaviors and interests (diagnostic criterion B). Social communication impairments are the hallmark of ASC, defined in terms of delay in social-emotional reciprocity and nonverbal-communication, and in developing and understanding social relationships (American Psychiatric Association, 2013). As in many other atypically developmental conditions (Marchetti et al., 2014; Lecciso et al., 2016), social communication impairments negatively impact the social functioning of individuals, as explained by the principles of the theory of mind (Baron-Cohen, 2000; Marchetti et al., 2014). To be specific, the deficit in the theory of mind, which is often called mindblindness (Lombardo and Baron-Cohen, 2011), leads children with an ASC to express difficulties in the understanding of the emotions of others that support their tendency of social withdrawal.

Several studies found a degree of delay in the development of emotional regulation functioning in individuals with an ASC, depending on IQ of children (Harms et al., 2010), in terms of facial emotion recognition [henceforth FER; (Hubert et al., 2007; Clark et al., 2008; Uljarevic and Hamilton, 2013; Lozier et al., 2014)] and facial emotion expression [henceforth FEE (Shalom et al., 2006; Zane et al., 2018; Capriola-Hall et al., 2019)]. These two competencies are often identified as being challenging areas for children with an ASC from the first years of life (Garon et al., 2009; Harms et al., 2010; Sharma et al., 2018) and may interfere with day-to-day social functioning even during later childhood and adulthood (Jamil et al., 2015; Cuve et al., 2018). Moreover, recognition and expression of emotions are two related competencies (Denham et al., 2003; Tanaka and Sung, 2016). During face-to-face interactions, an individual should capture the eye gaze of the other first to recognize the specific emotion he/she is expressing, and then to recreate it *via* an imitating process.

FER delay is related to eye avoidance (Kliemann et al., 2012; Grynszpan and Nadel, 2015; Sasson et al., 2016; Tanaka and Sung, 2016), which interferes with emotional processing and prevents individuals with an ASC from labeling the emotions. Regarding FEE, according to the simulation model (Illness SP-E in Mental, 2007), the delay is mainly related to the broken mirror neuron system (Williams et al., 2001; Rizzolatti et al., 2009), which prevents individuals with an ASC to mentally and physically recreate the observed action/emotion. In summary, individuals with an ASC show a delay in both emotional recognition and expression (Moody and Mcintosh, 2006; Ae et al., 2008; Iannizzotto et al., 2020a). To help them foster those competencies, forefront technology-based interventions have been developed (Scassellati et al., 2012; Grynszpan et al., 2014).

Within the research field of the Social Assistive Robotics system (Tapus et al., 2007; Feil-Seifer and Mataric, 2021), several technological devices have been designed to develop social skills in individuals with an ASC and promote the application of those devices as a daily life routine (Ricks and Colton, 2010). Interventions built based on those devices applied computer technology (Moore et al., 2000; Bernard-Opitz et al., 2001; Liu et al., 2008), robot systems (Dautenhahn and Werry, 2004; Kim et al., 2013; Lai et al., 2017), and virtual reality environments with an avatar (Conn et al., 2008; Welch et al., 2009; Bellani et al., 2011; Lahiri et al., 2011). This massive development in technological devices for the development of social skills in individuals with an ASC receives support from two recent theories on autism: the Intense World Theory by Markram and Markram (2010) and the Social Motivation Theory by Chevallier et al. (2012). According to the Intense World Theory (Markram and Markram, 2010), an autistic brain is constantly hyper-reactive and, as a consequence, perceptions and memories of environmental stimuli are memorized without filter. This continuous assimilation of information creates discomfort for individuals with an ASC who protect themselves by rejecting social interactions. The Social Motivation Theory (Chevallier et al., 2012) argued that individuals with an ASC are not prone to establish relationships with human partners, since they show a weak activation of the brain system in response to social reinforcements (Chevallier et al., 2012; Delmonte et al., 2012; Watson et al., 2015). This should explain the preference for the physical and mechanical world (Baron-Cohen, 2002). Technology-based types of training have the strength and potential to increase engagement and attention of children (Bauminger-Zviely et al., 2013), and to develop new desirable social behaviors (e.g., gestures, joint attention, spontaneous imitation, turn-taking, physical contact, and eye gaze) that are a prerequisite of the subsequent development of emotional competence (Robins et al., 2004; Zheng et al., 2014, 2016; So et al., 2016, 2019).

A huge amount of studies have already demonstrated that interventions applying technological devices have positive effects on the development of social functioning in individuals with an ASC (Liu et al., 2008; Diehl et al., 2012; Kim et al., 2013; Aresti-Bartolome and Garcia-Zapirain, 2014; Giannopulu et al., 2014; Laugeson et al., 2014; Peng et al., 2014; Vélez and Ferreiro, 2014; Pennisi et al., 2016; Hill et al., 2017; Kumazaki et al., 2017; Sartorato et al., 2017; Saleh et al., 2020). Most of the studies in this field adopted robots as social mediators (Diehl et al., 2012), playmates (Barakova et al., 2009), or as behavior-eliciting agents (Damianidou et al., 2020). Several studies reported that human-like robots are more engaging for individuals with an ASC than non-humanoid devices (Robins et al., 2004, 2006). Moreover, robots can engage individuals with an ASC during a task and reinforce their adequate behaviors (Scassellati, 2005; Freitas et al., 2017), since they are simpler, predictable, less stressful, and more consistent even compared with human-human interactions (Dautenhahn and Werry, 2004; Gillesen et al., 2011; Diehl et al., 2012; Yoshikawa et al., 2019).

Two very recent reviews (Damianidou et al., 2020; Saleh et al., 2020) considered studies applying robot-based training to improve social communication and interaction skills in individuals with an ASC. Only 6–10% of the studies reviewed by Damianidou et al. (2020) and Saleh et al. (2020) focused on emotion recognition and expression. Among those studies, four (Barakova and Lourens, 2010; Mazzei et al., 2012; Costa et al., 2013; Kim et al., 2017; Koch et al., 2017) were preliminary research on the software making the robots work; therefore, they did not directly test the effectiveness of the training. The FER ability was the focus of three studies (Costa et al., 2014; Koch et al., 2017; Yun et al., 2017). Costa et al. (2014), with an exploratory study, tested a robot-based intervention on two children with an ASC (age range = 14–16 years) and found an improvement in their ability to label emotions. The study by Koch et al. (2017) on 13 children with an ASC (age range = 5–11 years) compared a non-human-like robot-based intervention with a human-based one for FER ability. The level of engagement of the children was higher in the non-human-like robot-based intervention, and their behaviors were evaluated as more socially adequate than those of children trained with the human intervention. Finally, the study by Yun et al. (2017) applied a non-human-like robot-based compared to a similar human-based intervention on 15 children with an ASC (age range = 4–7 years) finding a general improvement in FER abilities of the children, but no differences between interventions.

On the other side, four studies have considered interventions for FEE abilities (Giannopulu and Pradel, 2012; Giannopulu et al., 2014; Bonarini et al., 2016; Soares et al., 2019). The study by Giannopulu and Pradel (Giannopulu and Pradel, 2012) is a single-case study examining the effectiveness of a non-human-like robot-based intervention on a child with a diagnosis of low-functioning autism (chronological age = 8 years; developmental age = 2 years). Training helped the child to use a robot as a mediator to initiate social interactions with humans and express emotions spontaneously. Giannopulu et al. (2014) compared a group of children with an ASC (*n* = 15) with a typically developing peer group (*n* = 20) with a mean age of 6–7 years old. Their findings showed that the children with an ASC, after the training, increased their emotional production, reaching the levels of the typically developing peers. Bonarini et al. (2016) applied a non-human-like robot-based intervention on three children with a low-functioning autism diagnosis (chronological age = 3 years; developmental age = not specified). They did not find any significant improvement.

Finally, Soares et al. (2019) compared three different conditions, intervention with a humanoid robot vs. intervention with a human vs. no intervention, on children with a diagnosis of high-functioning autism (*n* = 15 children for each group; age range = 5–10 years). They found that the children trained by the robot showed better emotion recognition and higher abilities to imitate facial emotion expressions compared with the other two groups.

Although these studies often do not use a randomized controlled trial experiment and their sample sizes are limited, their preliminary findings are still crucial for the development of research in this field. Technological-based interventions help individuals with an ASC to fill the gap and to overcome their delay in emotion recognition and expression. What is still under debate is whether the abilities acquired during the intervention with a robot are likely (or not) to be generalized in naturalistic interactions with human beings, as also requested in other conditions (Iannizzotto et al., 2020a,b; Pontikas et al., 2020; Valentine et al., 2020; Caprì et al., 2021). The direct generalization process from a robot-human interaction to a human-human interaction could be stressful for individuals with an ASC, because the stimuli produced by robots are simpler, predictable, less stressful, and more consistent than the ones produced by humans (Dautenhahn and Werry, 2004; Gillesen et al., 2011; Diehl et al., 2012; Yoshikawa et al., 2019). Therefore, intermediate and “hybrid” training that combines a technological device with the display of a human face of a peer, with standardized emotion expressions (Leo et al., 2018, 2019), could provide a fading stimulus to guide children with an ASC toward generalization of the acquired abilities. Such intermediate training should first be tested against the equivalent robot-based training to determine its efficacy and then can be used as a fading stimulus.

Albeit a previous systematic review (Ramdoss et al., 2012) argued that the evidence of computer-based interventions provided mixed results and highlighted critical issues, a recent meta-analysis (Kaur et al., 2013) reported that computer-based videos and games were used extensively and that they were useful in terms of improvement of social skills in children with an ASC. Despite contrasting conclusions, both the reviews suggested that further studies should be designed in order to better understand the critical issues of this kind of intervention.

This study places itself in this field of research to test a type of hybrid computer-based training with a standardized video of a peer compared with an equivalent robot-based intervention. To the best knowledge of the authors, this is the first attempt to test such intervention with children who are diagnosed with ASC. Specifically, we compared these two technological interventions to evaluate their effectiveness on the development of facial emotion recognition and expression abilities. We expected to find an overall significant difference between the pre- and post-interventions (i.e., HP1-time effect). In other words, we expected that recognition and expression abilities of children improved from pre- to post-interventions *via* the imitation process. Indeed, some evidence (Bandura, 1962; Bruner, 1974) highlighted that imitation is a key process to learn social skills, and it has been applied in other studies on children with autism (Zheng et al., 2014).

Two further research questions were formulated. RQ1-group effect: is there any difference in the emotion recognition and expression abilities between children who received a robot-based intervention and those who received a computer-based intervention (i.e., group effect)? RQ2-group^*^time effect: is there a significant interaction between type of intervention (i.e., group effect) and time of evaluations (i.e., time effect)?

A final research question considering engagement of children has been formulated. RQ3-engagement: we explored whether the hybrid technological device applied in this research induced a similar level of engagement compared with the humanoid robot. Previous studies (Dautenhahn and Werry, 2004; Diehl et al., 2012; Bauminger-Zviely et al., 2013; Yoshikawa et al., 2019) have compared the robot-child interaction with the child-human one; among them, only one (Yoshikawa et al., 2019) highlighted that the robot-child interaction is more engaging than the other. However, to the best knowledge of the authors, no studies have compared human-based intervention to computer-based intervention based on their level of engagement.

## Method and Materials

### Design and Procedure

A two-group pre-post-test study design (see Table 1) was applied to investigate the effectiveness of the two types of training conducted to develop and promote FEE of basic emotions (happiness, sadness, fear, and anger) in children with ASC. All the participants recruited in this study were diagnosed according to the gold standard measures (i.e., Autism Diagnostic Observation Schedule-2 and Autism Diagnostic Interview-Revised) and the Diagnostic and Statistical Manual of Mental Disorders, 5th Edition (DSM-5) diagnostic criteria. The diagnosis has been done by professionals working on two non-profit associations that helped us with the recruitment. These two associations are affiliated with the Italian National Health Service, and the severity of autistic traits is periodically evaluated in order to inform the psychological intervention provided by the service. The inclusion criterion was age range of children between 5 and 17 years; and the exclusion criteria were: (1) presence of comorbidity (2) lack of verbal ability, and (3) IQ below the normal range. Seventeen children met these criteria, and their families were invited to participate in the study. They received a brief description of the research protocol and then signed the informed consent. Data collection was performed in a quiet room in clinics where the associations have their headquarters. The Ethical Committee of the L'Adelfia non-profit association, which hosted the study, approved the research (01/2018) and informed consent was signed by the parents.

**Table 1:** Analytical description of the experimental design.

**Pre-intervention**	**Training using the technological devices**				**Post-intervention**
	**Day 1**	**Day 2**	**Day 3**	**Day 4**	
Evaluation of the children's facial emotion recognition and expression ability	Baseline on facial expression of emotion	Baseline on facial expression of emotion	Baseline on facial expression of emotion	Baseline on facial expression of emotion	Evaluation of the children's facial emotion recognition and expression ability
	1st session: H-S-F-A	1st session: S-F-A-H	1st session: F-A-H-S	1st session: A-H-S-F	
	2nd session: S-F-A-H	2nd session: F-A-H-S	2nd session: A-H-S-F	2nd session: H-S-F-A	
	3rd session: F-A-H-S	3rd session: A-H-S-F	3rd session: H-S-F-A	3rd session: S-F-A-H	
	4th session: A-H-S-F	4th session: H-S-F-A	4th session: S-F-A-H	4th session: F-A-H-S	
	Post-intervention on facial expression of emotion	Post-intervention on facial expression of emotion	Post-intervention on facial expression of emotion	Post-intervention on facial expression of emotion	

*H, happiness; S, sadness; F, fear; A, anger*.

**Figure 1:**
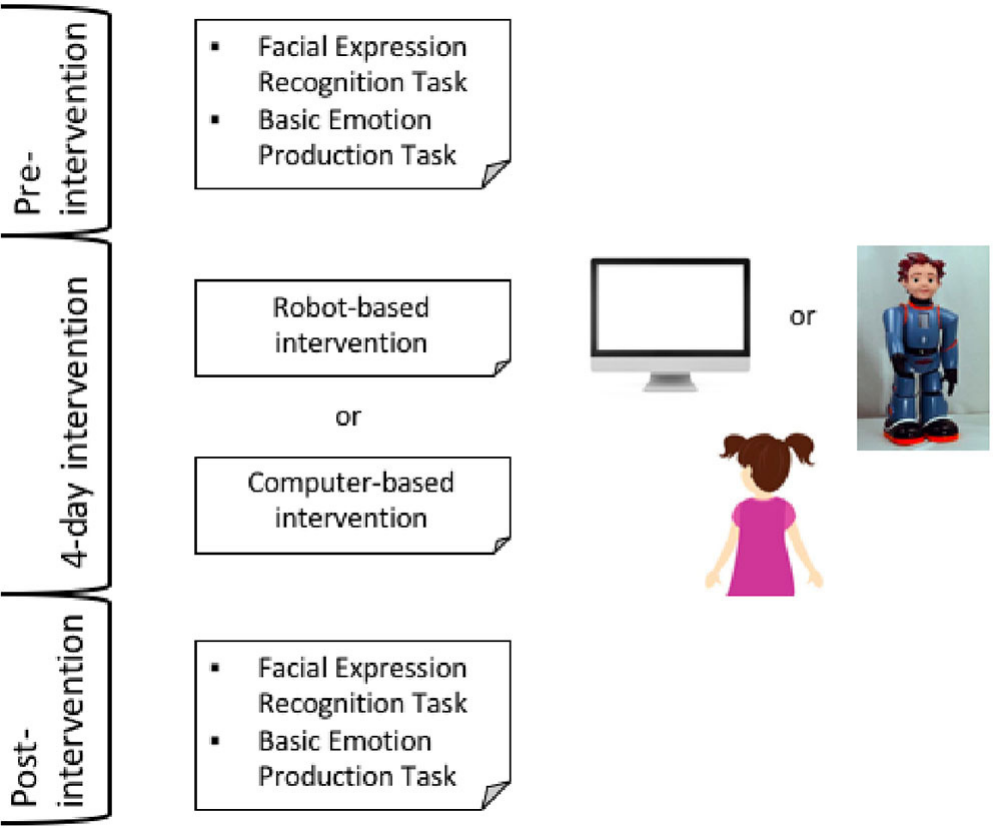
Diagram of the intervention phases and reproduction of the intervention settings.

Each child was first marched with a peer, creating a couple, with a similar chronological age (± 6 months) and IQ score (± 10 T-score) evaluated through the Raven Colored Progressive matrices (Measso et al., 1993). For five children, it was not possible to find a match with a similar age/IQ peer; therefore, they were not included in the study. Then, one child of the couple was assigned to one group and the other child to the other group. Table 1 and Figure 1 show the phases of the study. Both groups received a pre-intervention evaluation, such as measurement of FER and FEE abilities of children. The pre-intervention phase of the evaluation consisted of a 20-min session conducted in a quiet room by a trained therapist with the child seated in front of the therapist. The subsequent day (day 1 of treatment, see Table 1), one group (robot-intervention) received the training with the humanoid robot Zeno R25 [Robokind (Hanson et al., 2012; Cameron et al., 2016)], a device with a prerecorded childish voice (Matarić et al., 2007). The second group (computer-intervention) received the training with a video with a typically developing peer as a mediator. The training phase consisted of four days of intervention focused on the facial expression of basic emotions. Each day started with a baseline evaluation of the facial emotion expression ability during which the child was asked to express each basic emotion five times. Afterward, the training started with four sessions in which each emotion was expressed five times as a dynamic stimulus by the human-like robot and as a static stimulus in the intervention with the video. The child then had to imitate the expression five times. Each day of training ended with a post-intervention evaluation with a procedure similar to the one applied in the baseline evaluation at the beginning of the day. The emotion sequence was counterbalanced during the phase of the intervention (i.e., baseline, post-intervention, and training sessions). Finally, after 9–10 days, in the post-intervention, the therapist proposed the same evaluation done in the pre-intervention.

### Participants

Twelve out of 17 children with ASC (M = 9.33 years; sd = 2.19 years; range = 6–13 years; all males) were included in the study. Raven's Colored Progressive Matrices mean score was M = 105 (sd = 10.98). No significant differences were found in the chronological age and IQ scores between the two groups as well as in the pre-intervention evaluation. Most of the children were born at term (*n* = 10; 83.3%), one was pre-term; 58.3% of the children were first-born, and 16.6% of them were second-born or later; two children had a twin. All the children were enrolled in a behavioral intervention with the Applied Behavioral Analysis method. The mean age of the mother was 38.6 years (ds = 12.6 y), and their educational level was low (up to eight years of education) for 7.7%, intermediate (up to 13 years of education) for 30.8%, and high (15 or more years of education) for 84.6%. The mean age of father was 47.3 years (sd = 3.8 years), and their educational level was low (up to eight years of education) for 23.1%, intermediate (up to 13 years of education) for 53.8%, and high (15 or more years of education) for 15.4%. The parents were all married, except two who were divorced.

### Measures

Pre-intervention and post-intervention evaluations. To evaluate the ability of the children to recognize and express the four basic emotions (happiness, sadness, fear, and anger), we administered the Facial Emotion Recognition Task [FERT; adapted by Wang et al. (2011)] and the Basic Emotions Production Task (BEPT; technical report).

The Facial Emotion Recognition Task (FERT) is composed of 20 items (i.e., four emotions asked five times each). Each item included four black-and-white photographs of faces expressing the four basic emotions extracted by Ekman's FACS system (Ekman, 1984). The choice to include visual stimuli extracted from the FACS system is due to the fact that the software used in this study to evaluate the facial expressions of the children as correct or incorrect has been developed according to the FACS system and previously validated (Leo et al., 2018). In one example of the items, the therapist said to the child: “*Show me the happy face*.” The child, then, had to indicate the correct face among the four provided ones. The requests were provided sequentially to the child, as happiness-sadness-fear-anger, with no counterbalance. One point was attributed when the emotion was correctly detected and 0 points for wrong answers or no answers. One score for each emotion (range 0–5) and one total score were calculated as a sum of the correct answers (range 0–20).

The Basic Emotion Production task (BEPT; technical report) asked the child to express the four basic emotions without any external stimulus to imitate. For example, the therapist asked the child: “*Do you make me a happy face?*”. The requests were provided sequentially to the child as happiness-sadness-fear-anger, and the sequence was repeated five times. Each child was asked to express a total of 20 emotion expressions (four emotions ^*^ five times each). The facial expression of each child was scored as correct or incorrect by the software previously validated on typically and atypically developing children (Leo et al., 2018, 2019). One point was attributed when the emotion was correctly detected and 0 points for wrong answers or no answers. One score for each emotion (range 0–5) and one total score were calculated as a sum of the correct answers (range 0–20).

Interventions. The robot-intervention group received training with Zeno R25, a humanoid robot manufactured by Robokind (www.robokind.com). The robot has a face able to express emotions with seven degrees of freedom, such as eyebrows, mouth opening, and smile. The robot can also move its arms and legs. Zeno R25 features a system on a chip Texas Instruments OMAP 4460, OMAP 4460 dual-core 1.5 GHz ARM Cortex A9 processor with 1 GB of RAM and 16 GB of storage. The robot has Wi-Fi, Ethernet, two USB ports, an HDMI port, and an NFC chip for contactless data transfer. It is 56 cm tall and provided with sensors (gyroscope, accelerometer, compass, and infrared), a camera (five megapixels) lodged in his right eye, nine touch zones distributed over its entire skeleton, eight microphones, and a loudspeaker. On his chest, a 2.4-in LCD touch screen is used to access the functions and distribute content. The software part is based on the Ubuntu Linux distribution. The software includes basic software routines for invoking face and body movements. For the study purposes, an additional camera was placed on the robot chest (the same camera used for the second intervention group with the video instead of the robot). The camera was a full HD one (resolution 1,920 × 1,080 pixels), and it has been fixed at the height of the trouser belt of the robot (its least mobile part to reset the ego-motion). The robot has been connected *via* Ethernet with a laptop to which the additional camera has also been connected *via* USB. On the laptop, a software interface (GUI) properly built using the C++ environment and QT multiplatform library is installed. Through the interface, the commands to the robot are sent in order to invoke its speech and facial movement primitives. The images acquired from the camera were sent to the laptop *via* the USB connection, and they can be either stored (for subsequent processing) or also processed in real-time to provide immediate feedback to the child. When the child correctly answered the question, the robot would give him positive feedback (“Very well”); whereas if the child refused or did not correctly answer, the robot would continue with the task. The robot has a camera that follows the gaze of the child: if the child took his gaze off from the robot, he would receive a voice prompt made by the robot to engage him again in the task. The inputs of the voice prompt were given by the engineers who managed the software.

The second group of children was trained using pre-recorded videos of a typically developing peer performing facial expressions of emotions and reproducing the same procedure as done by the robot, such as the positive feedback (“Very well”). Similar to the robot-based intervention, in the computer-based training, the webcam of the computer followed the gaze of the children; if the child took his gaze off from the camera, he would receive a voice prompt made by the child/peer of the video. The inputs were given by the engineer who managed the software. The same GUI applied with Zeno has been used for this second intervention. In that case, the GUI sends commands to a video player with a playlist consisting of short videos of the typically developing peer. The child in the videos was trained by two of the authors of this study who are experts in developmental psychology. Each emotion expression was executed and recorded several times in order to have a range of videos among which choose the most appropriate ones. The same two experts selected a set of expressions performed according to the FACS principles (Ekman, 1984) and the GUI evaluated and chose for the training the ones that received the highest scores. The videos were projected on a 27-in monitor having full HD resolution. The monitor was placed on a cabinet, and at the bottom of the monitor, the same camera used for the sessions with the robot was placed. The software for automatic facial expression analysis running on the laptop was implemented using a C++ development environment also exploiting OpenCV (www.opencv.org) and OpenFace (github.com/TadasBaltrusaitis/OpenFace) libraries.

Engagement. The level of engagement was calculated as the number of voice prompts (i.e., the name of the child) that the two devices used to involve the child during the task. Each time the child took off his gaze from the device, the robot/peer would call the child by his name to engage him again in the task. The level of engagement ranged from 0 to 22 prompts (M = 4.5; sd = 6.7), with higher scores indicating lower engagement.

### Data Collection and Statistical Strategy

The videos were analyzed using modern computer vision technologies (Leo et al., 2020) specifically aimed for detecting and analyzing human faces for healthcare applications.

In particular, a type of software implemented elsewhere (Leo et al., 2018) and validated both on typically developing children and children with ASC (RStudio Team, 2020) was applied. The data were analyzed using RStudio Team (2020) and the Statistical Package for the Social Science v.25 (IBM Corp, 2010). In the pre-intervention, the competencies of the children on FER and BEP tasks were compared through independent sample t-tests. To test the hypothesis and the research questions, nonparametric analyses for longitudinal data on a small sample size were computed using the *nparLD* package (Noguchi et al., 2012) for RStudio. The F1LDF1 design was applied. The interventions (robot- vs. computer-based) were included as a group variable allowing the estimation of a group effect. The two evaluations (pre- and post-interventions) were included as a time variable allowing the estimation of a time effect. Finally, the interaction of group^*^time was included as well. The ANOVA-type test and the modified ANOVA-type test with box approximation were calculated for testing group effect, time effect, and their interaction. It is worth noting that the higher degree of freedom of each ANOVA model was equal to infinity, “in order to improve the approximation of the distribution under the hypothesis of ‘no treatment effects' and ‘no interaction between whole-plot factors'” [Noguchi et al., 2012, p. 14]. As a measure of the effect of the group^*^time interaction, we reported the relative treatment effect (RTE) ranging from 0 to 1 (Noguchi et al., 2012). When the interaction between group^*^time was significant, Mann–Whitney *U* was calculated. Bonferroni corrections have been applied. The Hedge's *g* effects size (Hedges and Olkin, 1985) has been calculated as well. A *p*-value of 0.05 was taken as statistically significant. A non-parametric Mann–Whitney test was carried out to evaluate whether the hybrid computer-based training is able to engage the attention of the child during the task similarly as the robot.

## Results

The results of the nonparametric longitudinal analyses are shown in Table 2 (for Facial Emotion Recognition Task) and Table 3 (for Basic Emotion Production Task). The modified ANOVA tests were not significant. Moreover, for both emotion recognition and expression scores, the ANOVA-type tests showed that significant group effects can be excluded (RQ1-group effect). This means that there were no significant differences between humanoid robot-based intervention and computer-based intervention on the facial emotion recognition and expression of the children. The facial recognition of happiness reached the ceiling (M = 5) in the pre-intervention evaluation in both groups; therefore, these scores have not been further analyzed.

**Table 2:** Results of the analyses for the FERT.

**Parameter**	**ANOVA-Type tests**			**Interactive effects**				**Hedges's *g* [LoCI, HiCI 95%]**	**Mann-Whitney *U***	**Bonferroni's correction**	**Mean(ds)**	
		***F* (df)**	***p***	**Robot-group**		**Computer-group**						
FERT sadness	Group	0.29 (1, ∞)	0.587	-		-			-	-	Robot-group: 4.83 (0.39)	Computer-group: 4.75 (0.45)
	Time	7.35 (1, ∞)	**0.006**	-		-			-	**0.003**	Pre-intervention: 4.58 (0.51)	Post-intervention: 5 (0)
	Group*Time	0.29 (1, ∞)	0.587	Robot*pre	0.44	Computer*pre	0.35		-	-	Robot-group Pre-intervention: 4.67 (0.52)	Computer-group Pre-intervention: 4.5 (0.55)
				Robot*post	0.60	Computer*post	0.60				Robot-group Post-intervention: 5 (0)	Computer-group Post-intervention: 5 (0)
Modified-ANOVA-Type test	*F*_(1, 9)_ = 0.294; *p* = 0.599							9.07 [7.14, 10.96]				
FERT fear	Group	1 (1, ∞)	0.317						-	-	Robot-group: 4.83 (0.58)	Computer-group: 5 (0)
	Time	1 (1, ∞)	0.317						-	-	Pre-intervention : 4.83 (0.58)	Post-intervention : 5 (0)
	Group*Time	1 (1, ∞)	0.317	Robot*pre	0.44	Computer*pre	0.52		-	-	Robot-group Pre-intervention : 4.67 (0.82)	Computer-group Pre-intervention : 5 (0)
				Robot*post	0.52	Computer*post	0.52				Robot-group Post-intervention: 5 (0)	Computer-group Post-intervention : 5 (0)
Modified- ANOVA-Type test	*F*_(1, 5)_ = 1; *p* = 0.363							9.40 [7.40, 11.34]				
FERT anger	Group	1 (1, ∞)	0.317						-	-	Robot-group: 5 (0)	Computer-group: 4.92 (0.29)
	Time	1 (1, ∞)	0.317						-	-	Pre-intervention: 4.92 (0.29);	Post-intervention: 5 (0).
	Group*Time	1 (1, ∞)	0.317	Robot*pre	0.52	Computer*pre	0.44		-	-	Robot-group Pre-intervention: 5 (0)	Computer-group Pre-intervention: 5 (0)
				Robot*post	0.52	Computer*post	0.52				Robot-group Post-intervention : 5 (0)	Computer-group Post-intervention: 5 (0)
Modified- ANOVA-Type test	*F*_(1, 5)_ = 1; *p* = 0.363							11.28 [8.91, 13.58]				
FERT total score	Group	0.821 (1, ∞)	0.364						-	-	Robot-group: 19.67 (0.89);	Computer-group: 19.67 (0.49).
	Time	10.636 (1, ∞)	**0.001**						-	**0.000**	Pre-intervention: 19.33 (0.89);	Post-intervention: 20 (0)
	Group*Time	0.821 (1, ∞)	0.364	Robot x pre	0.44	Computer x pre	0.30		-	-	Robot-group Pre-intervention: 19.33 (1.21);	Computer-group Pre-intervention: 19.33 (0.52);
				Robot x post	0.62	Computer x post	0.62				Robot-group Post-intervention: 20 (0).	Computer-group Post-intervention: 20 (0).
Modified- ANOVA-Type test	*F*_(1, 9)_ = 0.820; *p* = 0.386							30.71 [24.24, 36.85]				

FERT, facial emotion recognition task; group, robot- vs. computer-based intervention; time, pre- vs. post-test; group*time, interaction of intervention group and time of evaluation. Bold indicates significant results.

**Table 3:** Results of the analyses for the BEPT.

				**Relative Treatment Effects (RTE)**				**Hedges's *g* [LoCI; HiCI 95%]**	**Mann-Whitney *U***	**Bonferroni correction**	**Mean(ds)**	
**Parameter**	**ANOVA-Type tests**			**Interactive effects**								
		***F***	***p*-value**	**Robot-group**		**Computer-group**						
BEPT happiness	Group	2.414 (1, ∞)	0.120						-		Robot-group: 4.50 (1)	Computer-group: 3 (2.17)
	Time	5.720 (1, ∞)	**0.016**							**0.008**	Pre-intervention: 3.25 (1.81)	Post-intervention: 4.25 (1.76).
	Group*Time	0.082 (1, ∞)	0.774	Robot*pre	0.49	Computer*pre	0.33				Robot-group Pre-intervention: 4 (1.26)	Computer-group Pre-intervention: 2.5 (2.07)
				Robot*post	0.69	Computer*post	0.49				Robot-group Post-intervention: 5 (0)	Computer-group Post-intervention: 3.50 (2.34)
Modified- ANOVA-Type test	*F*_(1, 6)_ = 2.414; *p* = 0.166							2.39 [1.64, 3.12]				
BEPT sadness	Group	1.460 (1, ∞)	0.226								Robot-group: 2.92 (1.78);	Computer-group: 2 (1.86).
	Time	10.899 (1, ∞)	**0.001**							**0.000**	Pre-intervention: 1.50 (1.32)	Post-intervention: 3.42 (1.78)
	Group*Time	0.001 (1, ∞)	0.970	Robot*pre	0.42	Computer*pre	0.27				Robot-group Pre-intervention: 2 (1.67)	Computer-group Post-intervention: 1 (0.89)
				Robot*post	0.72	Computer*post	0.59				Robot-group Post-intervention: 3.83 (1.47)	Computer-group Post-intervention: 3 (2.1)
Modified- ANOVA-Type test	*F*_(1, 9)_ = 1.460; *p* = 0.255							1.43 [0.79, 2.05]				
BEPT fear	Group	0.000 (1, ∞)	NA						-		Robot-group: 1.83 (1.75)	Computer-group: 2.08 (2.19)
	Time	30.518 (1, ∞)	**<0.0001**						-	**<0.0001**	Pre-intervention: 0.92 (1.38)	Post-intervention: 3 (1.91)
	Group*Time	1.019 (1, ∞)	**0.031**	Robot*pre	0.37	Computer*pre	0.31		*U* = 14.000, *p* = 0.503	**0.015**	Robot-group Pre-intervention: 1 (1.26)	Computer-group Pre-intervention: 0.83 (1.6)
				Robot*post	0.63	Computer*post	0.69				Robot-group Pre-intervention: 2.67 (1.86).	Computer-group Post-intervention: 3.33 (2.07).
Modified- ANOVA-Type test	*F*_(1, 9)_ = 0; *p* = NA							1.01 [0.41, 1.60]				
BEPT anger	Group	0.189 (1, ∞)	0.066						-	-	Robot-group: 3.17 (1.75)	Computer-group: 2.75 (2.26)
	Time	24.997 (1, ∞)	**<0.0001**						-	**<0.0001**	Pre-intervention: 1.83 (1.59)	Post-intervention: 4.08 (1.73)
	Group*Time	1.039 (1, ∞)	**0.031**	Robot*pre	0.38	Computer*pre	0.26		*U* = 16.000; *p* = 0.673	**0.015**	Robot-group Pre-intervention: 2.33 (1.63)	Computer-group Pre-intervention: 1.33 (1.51)
				Robot*post	0.67	Computer*post	0.69				Robot-group Post-intervention: 4 (1.55)	Computer-group Post-intervention: 4.17 (2.04)
Modified- ANOVA-Type test	*F*_(1, 9)_ = 0.189; *p* = 0.672							1.67 [1.01, 2.31]				
BEPT total score	Group	0.852 (1, ∞)	0.355					-	-	-	Robot-group: 13.08 (4.81)	Computer-group: 10.42 (6.54)
	Time	15.101 (1, ∞)	**<0.0001**					-	-	**<0.0001**	Pre-intervention: 8.75 (4.94)	Post-intervention: 14.75 (5.08)
	Group*Time	0.337 (1, ∞)	0.561	Robot*pre	0.43	Computer*pre	0.27	-	-	-	Robot-group Pre-intervention: 10.67 (4.37)	Computer-group Pre-intervention: 6.83 (5.08)
				Robot*post	0.69	Computer*post	0.61				Robot-group Post-intervention: 15.50 (4.23)	Computer-group Post-intervention: 14 (6.13)
Modified- ANOVA-Type test	*F*_(1, 9)_ = 0.852; *p* = 0.377							2.70 [1.91, 3.47]			

BEPT, basic emotion expression task; group, robot- vs. computer-based intervention; time, pre- vs. post-test; group*time, interaction of intervention group and time of evaluation. Bold indicates significant results.

The results of the time effects and group^*^time effects revealed several significance. Regarding the FERT (see Table 2), significant results emerged in the time effect of sadness with post-evaluation scores higher than those of pre-evaluation scores (HP1-time effect). Similarly, the results revealed a time effect for the FERT total score mining that all the children improved their broader ability to recognize basic emotions when they were trained by a technological device. Regarding the BEPT, significant time effects emerged for all the four basic emotions and for the BEPT total score, with scores in the post-intervention always higher than the scores in the pre-intervention (HP1-time effect). This means that the children acquired higher performances in the expression of basic emotions after interventions with the technological devices. Regarding the expression of fear and anger, the ANOVA-type tests showed two significant effects for the group^*^time interaction. However, the Mann–Whitney tests did not find a significant difference among the four subgroups. This corroborated the idea that both interventions (robot and computer) improved the ability of the children (RQ2-group^*^time effect).

The comparison of the level of engagement during the two training sessions showed no significant difference (*U* = 13.000; *p* =0.413). This means that the hybrid technological device applied in this research induced a similar level of engagement compared with the humanoid robot (RQ3-engagement).

## Discussion

The main study purpose was to give a contribution to the field of research regarding the application of technology to improve the emotional competencies of individuals with an ASC. In particular, the main focus was on whether the proposed computer-based intervention would be effective in terms of the development and promotion of facial emotion recognition and expression. We debated that a straightforward generalization, from the technological device to the human interaction, might be stressful for individuals with an ASC, and that an intermediate transition with hybrid training would help the generalization process. For this reason, this study presented a two-group pre-post-test study design testing the effectiveness of two technological-based interventions aimed at developing facial emotion expression and recognition in children with an ASC. The technology on which the interventions are based exploited a robot and a pre-recorded video with a typically developing peer.

The first hypothesis expected to find an overall significant difference between the pre- and post-intervention evaluation phases demonstrating that the interventions improved facial emotion expression and recognition abilities of children. The expression and recognition of four basic emotions (happiness, sadness, fear, and anger) were considered in two groups of 12 children with an ASC. The results corroborated the preliminary hypothesis revealing an improvement in the broader ability to recognize and express basic emotions. Moreover, the findings showed a higher post-intervention recognition of the negative emotion of sadness and higher post-intervention production of happiness, sadness, fear, and anger. Albeit the study limitation is related to sample size, this evidence is in line with previous studies (Pennisi et al., 2016; Hill et al., 2017; Kumazaki et al., 2017; Sartorato et al., 2017; Saleh et al., 2020) suggesting that intensive training that applies technological devices helps children in filling the gap.

The study also proposed three research questions. First of all, this study compared the efficacy of the two interventions (robot vs. computer) on basic emotion recognition and expression (RQ1-group effect). The findings revealed that a group effect can be excluded: this means that there was no difference in the performance of the children with the two technological devices. In other words, the application of technology itself, as previously discussed, not the type of technology applied, fosters improvement. This is the first attempt to evaluate the effectiveness of the two interventions promoting emotional competence comparing two different technological devices; therefore, the preliminary results need further demonstration with a larger sample.

The second research question (RQ2-group^*^time effect) asked whether there was a significant interaction between the two technological-based interventions (robot- vs. computer-based) and the two times of evaluation (pre- vs. post-test). The results showed a significant interaction effect regarding the expression of fear and anger. However, further comparisons with Mann–Whitney *U* were not significant.

Finally, we investigated whether the hybrid computer training had a similar level of engagement compared with the robot. The exploratory evidence suggested no difference in the levels of engagement, considered in the form of the number of voice prompts given by the device, between children trained by the robot and those trained by the computer. In other words, the engagement degrees of the children were pretty high, as demonstrated by the low mean, and similar across the two devices.

Therefore, albeit the results in this study should be interpreted cautiously, they provided the first evidence supporting the use of hybrid technology as a mediator to facilitate and smoothen the processing of emotions in the human face by individuals with an ASC, similar to the findings of Golan et al. (2010) who used a video displaying a human face of an adult. An intermediate and “hybrid” type of training that combines a technological device with the display of a human face, with standardized emotion expressions, may provide a fading stimulus to guide children with an ASC toward generalization of acquired abilities. Future studies should test and validate the hybrid training with a larger sample and test whether its effectiveness in guiding children toward the generalization of emotion recognition and expression from the robot, to the hybrid device, to the human face.

## Limitations

This study presents some limitations. The small sample size, although similar to other studies in the same field, limited the breadth of the conclusions. Future research should test the effectiveness of the two interventions with a larger sample size. Although the two groups of children were matched according to chronological and mental age and they are not significantly different based on their baseline evaluations, we suggest that the wide age range represents a limitation for this study. The second limitation is linked to the lack of information regarding psychological parameters other than age and IQ, such as the severity of autistic traits and information on general, social functioning, and adaptive behaviors. Because of privacy concerns, it was not possible to have this information. Finally, the third limitation concerns the lack of the wait-list control group of children who did not receive any intervention. In order to test whether the improvement in emotional skills of the children depended on the technological-based interventions, further study should be designed with a wait-list control group.

## Future Direction

The evidence demonstrated the effectiveness of training on emotion recognition and expression when a technological device is used as a mediator. The data confirmed the benefit produced by training mediated by a humanoid robot and, concurrently, a similar impact when a hybrid device is used. Furthermore, the data showed a similar level of engagement of the children with the robot and the video on the computer. Therefore, a further step in this field would be the implementation of a research plan considering a repeated measure design with three phases, starting from intensive robot-based training, followed by the first generalization with hybrid computer-based training, and then by the full generalization of acquired skills in naturalistic settings toward adults and peers.

## Data Availability Statement

The data that support the findings of this study are available from the corresponding author, upon reasonable request.

## Ethics Statement

The studies involving human participants were reviewed and approved by L'Adelfia non-profit association ethical committee. Written informed consent to participate in this study was provided by the participants' legal guardian/next of kin.

## Author Contributions

SP and FL conceived the study, and together with AL developed the design. AL recruit participants and together with ML, PC, PS, and PM collected data. AL, RF, and TC carried out the statistical analysis. ML, CD, PC, PM, and PS developed the technological devices, the softwares and analyzed data collected by the robot and the computer-based application. FL, AL, and SP wrote the draft paper. ML wrote the technical section of the robot and computer/video intervention. All authors read the paper, gave their feedback, and approved the final version.

## Conflict of Interest

The authors declare that the research was conducted in the absence of any commercial or financial relationships that could be construed as a potential conflict of interest.
